# Quality of life and fear of death among patients living with HIV during the COVID-19 pandemic second wave

**DOI:** 10.1192/j.eurpsy.2023.1719

**Published:** 2023-07-19

**Authors:** V. V. Titova, V. I. Rozhdestvenskiy, I. A. Gorkovaya, D. O. Ivanov, Y. S. Aleksandrovich

**Affiliations:** 1 Department of Psychosomatics and Psychotherapy, Saint Petersburg State Pediatric Medical University, Saint Petersburg, Russian Federation

## Abstract

**Introduction:**

The new coronavirus pandemic has brought the issue of life quality to the forefront. Among HIV-infected patients, life quality may be associated with fear of death.

**Objectives:**

The study aimed to investigate the life quality and death fear among HIV-infected patients during the pandemic second wave in Russia.

**Methods:**

The data were collected from February to July 2021 using a Google form that we developed. Fifty-nine patients living with HIV participated in the study. We used the WHOQOL-BREF to examine the quality of life and the Fear of Personal Death Scale to explore fear of death. Both questionnaires were adapted for use in Russia.

**Results:**

We found the following mean values for the domains: “physical and psychological well-being” — M = 21.39±3.61; “self-perception” — M = 17.51±2.28; “microsocial support” — M = 9.15±2.94; “social well-being” — M = 24.81±5.33. We found that physical and psychological well-being were associated with the transcendental consequences of death (r_xy_ = 0.274, p < 0.05), self-perception with body consequences (r_xy_ = -0.304, p < 0.05) and fear of forgetting (r_xy_ = -0.287, p < 0.05), and social well-being with body consequences (r_xy_ = -0.310, p < 0.05).

**Conclusions:**

Life quality is related to intrapersonal, interpersonal, and supra-personal dimensions of death fear during the second wave of the pandemic among patients living with HIV. Such fact may indicate possible psychotherapeutic targets when working with this group of patients.

**Disclosure of Interest:**

None Declared

